# Supplementary material for: Constitutive OsCIN1 Expression Reprograms Source–Sink Dynamics and Compromises Agronomic Traits in Rice
Source: Int J Mol Sci. 2025 Nov 27;26(23):11471. doi: 10.3390/ijms262311471 (PMC12692661; doi:10.3390/ijms262311471)
Supplement: Supplementary file 1 [file ijms-26-11471-s001.zip › ijms-3955025-supplementary.pdf]

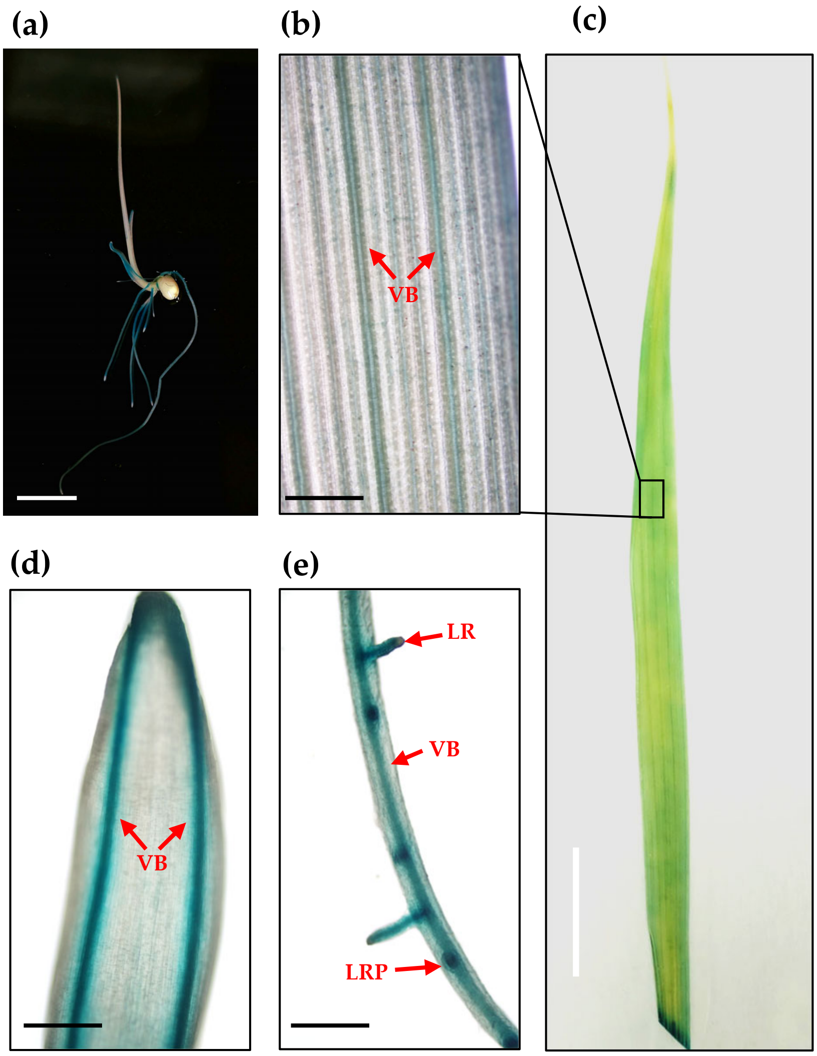

Supplementary Figure S1. Tissue-specific expression pattern of pOsCIN1::GUS in rice. **(a)** GUS expression in the 5-day-old seedling. **(b)** Leaf section under microscope. **(c)** Full expanded leaf of 2-week-old plant with GUS staining (left) and no staining (right). **(d,e)** GUS expression under microscope of **(d)** coleoptile, **(e)** root. VS: vascular bundle; LR: lateral root; LRP: lateral root primordium. Scale bars (a,c): 1 cm, (b,d,e): 20 μm.
